# 2-Amino-3-ammonio­pyridinium dichloride

**DOI:** 10.1107/S1600536810003624

**Published:** 2010-02-03

**Authors:** Madhukar Hemamalini, Hoong-Kun Fun

**Affiliations:** a X-ray Crystallography Unit, School of Physics, Universiti Sains Malaysia, 11800 USM, Penang, Malaysia

## Abstract

The asymmetric unit of the title compound, C_5_H_9_N_3_^2+^·2Cl^−^, contains two diprotonated 2,3-diamino­pyridine cations and four chloride anions. In the crystal structure, the anions and cations are connected by inter­molecular N—H⋯Cl and C—H⋯Cl hydrogen bonds, forming a three-dimensional network. The crystal structure is further stabilized by π–π inter­actions between pyridinium rings [centroid–centroid distance = 3.695 (1) Å].

## Related literature

For background to the chemistry of substituted pyridines and chloride anions, see: Pozharski *et al.* (1997 ▶); Katritzky *et al.* (1996 ▶); Abu Zuhri & Cox (1989 ▶); De Cires-Mejias *et al.* (2004 ▶); Sessler *et al.* (2003 ▶). For related structures, see: Fun & Balasubramani (2009 ▶); Balasubramani & Fun (2009*a* ▶,*b* ▶). For details of hydrogen bonding, see: Jeffrey & Saenger (1991 ▶); Jeffrey (1997 ▶); Scheiner (1997 ▶). For reference bond-length data, see: Allen *et al.* (1987 ▶). For the stability of the temperature controller used in the data collection, see: Cosier & Glazer (1986 ▶).
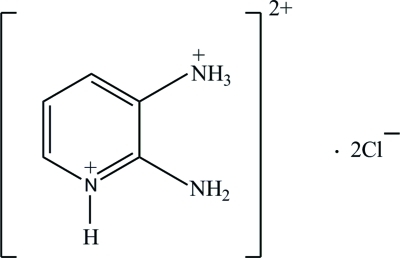


## Experimental

### 

#### Crystal data

C_5_H_9_N_3_^2+^·2Cl^−^
*M* *_r_* = 182.05
Monoclinic, 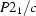

*a* = 10.9770 (2) Å
*b* = 12.5175 (2) Å
*c* = 11.6520 (2) Å
β = 98.979 (1)°
*V* = 1581.42 (5) Å^3^
*Z* = 8
Mo *K*α radiation
μ = 0.75 mm^−1^
*T* = 100 K
0.34 × 0.32 × 0.13 mm

#### Data collection

Bruker APEX DUO CCD area-detector diffractometer
Absorption correction: multi-scan (*SADABS*; Bruker, 2009 ▶) *T*_min_ = 0.787, *T*_max_ = 0.907
22610 measured reflections
5736 independent reflections
4042 reflections with *I* > 2σ(*I*)
*R*_int_ = 0.035

#### Refinement

*R*[*F* ^2^ > 2σ(*F* ^2^)] = 0.045
*wR*(*F* ^2^) = 0.109
*S* = 1.03
5736 reflections
253 parameters
H atoms treated by a mixture of independent and constrained refinement
Δρ_max_ = 0.44 e Å^−3^
Δρ_min_ = −0.22 e Å^−3^

### 

Data collection: *APEX2* (Bruker, 2009 ▶); cell refinement: *SAINT* (Bruker, 2009 ▶); data reduction: *SAINT*; program(s) used to solve structure: *SHELXTL* (Sheldrick, 2008 ▶); program(s) used to refine structure: *SHELXTL*; molecular graphics: *SHELXTL*; software used to prepare material for publication: *SHELXTL* and *PLATON* (Spek, 2009 ▶).

## Supplementary Material

Crystal structure: contains datablocks global, I. DOI: 10.1107/S1600536810003624/wn2374sup1.cif

Structure factors: contains datablocks I. DOI: 10.1107/S1600536810003624/wn2374Isup2.hkl

Additional supplementary materials:  crystallographic information; 3D view; checkCIF report

## Figures and Tables

**Table 1:** Hydrogen-bond geometry (Å, °)

*D*—H⋯*A*	*D*—H	H⋯*A*	*D*⋯*A*	*D*—H⋯*A*
N1—H1*N*1⋯Cl2^i^	0.84 (2)	2.26 (2)	3.081 (2)	164.4 (18)
N1—H2*N*1⋯Cl1^ii^	0.87 (2)	2.27 (2)	3.114 (1)	167 (2)
N1—H3*N*1⋯Cl4^iii^	0.89 (2)	2.47 (2)	3.223 (2)	143.2 (18)
N2—H1*N*2⋯Cl1^iv^	0.90 (2)	2.32 (2)	3.219 (2)	177.6 (16)
N2—H2*N*2⋯Cl2^v^	0.79 (2)	2.59 (2)	3.242 (2)	142 (2)
N3—H1*N*3⋯Cl2	0.82 (2)	2.26 (2)	3.060 (2)	166 (2)
N4—H1*N*4⋯Cl3^vi^	0.91 (2)	2.17 (2)	3.049 (2)	163.2 (17)
N4—H2*N*4⋯Cl4	0.93 (2)	2.77 (2)	3.454 (2)	131.5 (17)
N4—H2*N*4⋯Cl1^vi^	0.93 (2)	2.51 (2)	3.148 (2)	126.4 (18)
N4—H3*N*4⋯Cl4^vii^	0.83 (2)	2.30 (2)	3.128 (2)	175 (2)
N5—H1*N*5⋯Cl4	0.91 (2)	2.29 (2)	3.193 (2)	172.0 (18)
N5—H2*N*5⋯Cl1^viii^	0.84 (2)	2.77 (2)	3.340 (2)	126.6 (17)
N6—H1*N*6⋯Cl3	0.89 (2)	2.22 (2)	3.057 (1)	156.6 (19)
C7—H7*A*⋯Cl4^ix^	0.99 (2)	2.745 (19)	3.459 (2)	129.4 (14)

Symmetry codes: (i) 
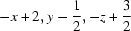
; (ii) 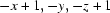; (iii) 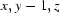; (iv) 
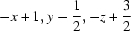
; (v) 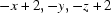; (vi) 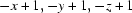; (vii) 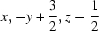; (viii) 
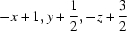
; (ix) 
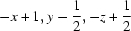
.

**Fig. 1.**
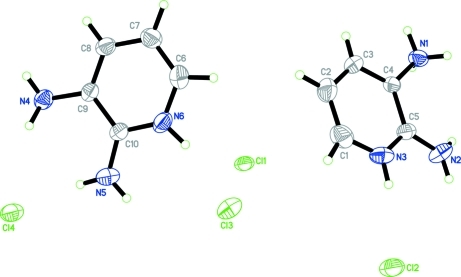
The asymmetric unit of the title compound. Displacement ellipsoids are drawn at the 50% probability level. Hydrogen atoms are shown as spheres of arbitrary radius.

**Fig. 2.**
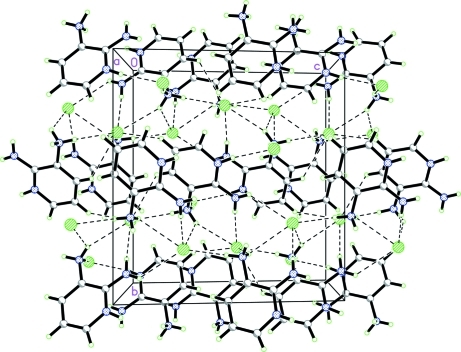
The crystal packing of the title compound, showing the hydrogen-bonded (dashed lines) networks.

## supplementary crystallographic information

### Comment

Pyridine and its derivatives play an important role in heterocyclic chemistry (Pozharski *et al.*, 1997; Katritzky *et al.*, 1996). In particular, diaminopyridines play an important role in the preparation of aromatic azo dyes, the subject of many polarographic investigations (Abu Zuhri & Cox, 1989). The coordination chemistry of anions is a fast-growing area of supramolecular chemistry. Moreover, Cl anions have been successfully used to assemble double-helical motifs of various molecules containing aromatic groups, with stacking within the helices (Sessler *et al.*, 2003). Pyridine and its substituted derivatives are often involved in hydrogen-bond interactions (Jeffrey & Saenger, 1991; Jeffrey, 1997; Scheiner, 1997).  The crystal structures of 2,3-diaminopyridinium 4-hydroxybenzoate (Fun & Balasubramani, 2009), 2,3-diaminopyridinium 4-nitrobenzoate (Balasubramani & Fun, 2009*a*) and 2,3-diaminopyridinium benzoate (Balasubramani & Fun, 2009*b*) have recently been reported by us. In the hope of studying some interesting hydrogen-bonding interactions, the title compound was synthesized. Its molecular and crystal structure is presented here.

The asymmetric unit of the title compound (Fig. 1) consists of two diprotonated 2,3-diaminopyridine cations and four chloride anions. In the 2,3-diaminopyridinium cations, protonation at atoms N3 and N6 has led to slight increases in the C1—N3—C5  and C6—N6—C10 angles to 123.65 (14)° and 123.92 (14)°, respectively, compared to those of an unprotonated structure (De Cires-Mejias *et al.*, 2004). The bond lengths (Allen *et al.*, 1987) and angles are normal .

In the crystal structure (Fig. 2), the anions and cations are connected by intermolecular N—H···Cl and C—H···Cl hydrogen bonds, forming a three-dimensional network.  The crystal structure is further stabilized by π···π interactions between the pyridinium rings (N3/C1–C5) [centroid-to-centroid (2-x, -y, 1-z) distance = 3.695 (1) Å].

### Experimental

To a hot methanol solution (20 ml) of 2,3-diaminopyridine (27 mg, Aldrich) was added a few drops of hydrochloric acid. The solution was warmed over a water bath for a few minutes. The resulting solution was allowed to cool slowly to room temperature. Crystals of the title compound appeared from the mother liquor after a few days.

### Refinement

All the H atoms were located in a difference Fourier map and allowed to refine freely [N—H = 0.82 (2) - 0.93 (3) Å, C—H = 0.93 (2) - 1.00 (2) Å].

### Crystal data

**Table:**

C_5_H_9_N_3_^2+^·2Cl^−^	*F*(000) = 752
*M**_r_* = 182.05	*D*_x_ = 1.529 Mg m^−^^3^
Monoclinic, *P*2_1_/*c*	Mo *K*α radiation, λ = 0.71073 Å
Hall symbol: -P 2ybc	Cell parameters from 4939 reflections
*a* = 10.9770 (2) Å	θ = 2.4–31.0°
*b* = 12.5175 (2) Å	µ = 0.75 mm^−^^1^
*c* = 11.6520 (2) Å	*T* = 100 K
β = 98.979 (1)°	Block, brown
*V* = 1581.42 (5) Å^3^	0.34 × 0.32 × 0.13 mm
*Z* = 8	

### Data collection

**Table:**

Bruker APEX DUO CCD area-detector diffractometer	5736 independent reflections
Radiation source: fine-focus sealed tube	4042 reflections with *I* > 2σ(*I*)
graphite	*R*_int_ = 0.035
φ and ω scans	θ_max_ = 32.6°, θ_min_ = 1.9°
Absorption correction: multi-scan (*SADABS*; Bruker, 2009)	*h* = −16→15
*T*_min_ = 0.787, *T*_max_ = 0.907	*k* = −18→18
22610 measured reflections	*l* = −17→17

### Refinement

**Table:**

Refinement on *F*^2^	Primary atom site location: structure-invariant direct methods
Least-squares matrix: full	Secondary atom site location: difference Fourier map
*R*[*F*^2^ > 2σ(*F*^2^)] = 0.045	Hydrogen site location: inferred from neighbouring sites
*wR*(*F*^2^) = 0.109	H atoms treated by a mixture of independent and constrained refinement
*S* = 1.02	*w* = 1/[σ^2^(*F*_o_^2^) + (0.0489*P*)^2^ + 0.1879*P*] where *P* = (*F*_o_^2^ + 2*F*_c_^2^)/3
5736 reflections	(Δ/σ)_max_ = 0.001
253 parameters	Δρ_max_ = 0.44 e Å^−^^3^
0 restraints	Δρ_min_ = −0.22 e Å^−^^3^

### Special details

**Table:**

Experimental. The crystal was placed in the cold stream of an Oxford Cryosystems Cobra open-flow nitrogen cryostat (Cosier & Glazer, 1986) operating at 100.0 (1) k.
Geometry. All s.u.'s (except the s.u. in the dihedral angle between two l.s. planes) are estimated using the full covariance matrix. The cell s.u.'s are taken into account individually in the estimation of s.u.'s in distances, angles and torsion angles; correlations between s.u.'s in cell parameters are only used when they are defined by crystal symmetry. An approximate (isotropic) treatment of cell s.u.'s is used for estimating s.u.'s involving l.s. planes.
Refinement. Refinement of F^2^ against ALL reflections. The weighted R-factor wR and goodness of fit S are based on F^2^, conventional R-factors R are based on F, with F set to zero for negative F^2^. The threshold expression of F^2^ > 2σ(F^2^) is used only for calculating R-factors(gt) etc. and is not relevant to the choice of reflections for refinement. R-factors based on F^2^ are statistically about twice as large as those based on F, and R- factors based on ALL data will be even larger.

### Fractional atomic coordinates and isotropic or equivalent isotropic displacement parameters (Å^2^)

**Table:**

	*x*	*y*	*z*	*U*_iso_*/*U*_eq_	
Cl1	0.28885 (4)	0.19696 (4)	0.71503 (3)	0.03796 (11)	
Cl2	1.04311 (4)	0.14551 (4)	0.94363 (3)	0.03993 (12)	
Cl3	0.78343 (4)	0.38414 (4)	0.69663 (3)	0.04112 (12)	
Cl4	0.47897 (4)	0.81032 (4)	0.52864 (3)	0.04069 (12)	
N1	0.77439 (14)	−0.16705 (12)	0.55300 (12)	0.0311 (3)	
N2	0.89490 (15)	−0.10343 (15)	0.77888 (13)	0.0415 (4)	
N3	0.94910 (13)	0.05666 (13)	0.70138 (12)	0.0364 (3)	
C1	0.94453 (17)	0.13025 (16)	0.61525 (17)	0.0413 (4)	
C2	0.88294 (17)	0.10955 (15)	0.50822 (16)	0.0408 (4)	
C3	0.82455 (15)	0.01104 (14)	0.48808 (13)	0.0323 (3)	
C4	0.82915 (13)	−0.06214 (12)	0.57545 (12)	0.0255 (3)	
C5	0.89151 (13)	−0.03864 (13)	0.68821 (12)	0.0279 (3)	
N4	0.49075 (14)	0.64395 (13)	0.29409 (13)	0.0327 (3)	
N5	0.62585 (16)	0.59088 (15)	0.52171 (13)	0.0409 (4)	
N6	0.70640 (13)	0.44374 (12)	0.44118 (12)	0.0345 (3)	
C6	0.71801 (16)	0.37487 (15)	0.35421 (16)	0.0384 (4)	
C7	0.65521 (16)	0.39087 (15)	0.24616 (15)	0.0366 (4)	
C8	0.57784 (15)	0.48000 (14)	0.22721 (13)	0.0315 (3)	
C9	0.56779 (13)	0.54973 (13)	0.31554 (12)	0.0269 (3)	
C10	0.63317 (13)	0.53062 (13)	0.42861 (12)	0.0276 (3)	
H1A	0.9841 (18)	0.1899 (18)	0.6438 (18)	0.054 (6)*	
H2A	0.8819 (18)	0.1582 (18)	0.4467 (18)	0.051 (6)*	
H3A	0.7863 (16)	−0.0052 (15)	0.4205 (15)	0.032 (5)*	
H6A	0.7766 (17)	0.3164 (17)	0.3824 (17)	0.051 (6)*	
H7A	0.6660 (17)	0.3404 (17)	0.1831 (16)	0.045 (5)*	
H8A	0.5334 (16)	0.4917 (14)	0.1540 (14)	0.031 (4)*	
H1N1	0.8271 (19)	−0.2160 (18)	0.5682 (17)	0.048 (6)*	
H2N1	0.744 (2)	−0.1729 (19)	0.480 (2)	0.063 (7)*	
H3N1	0.705 (2)	−0.1742 (19)	0.5822 (19)	0.063 (7)*	
H1N2	0.845 (2)	−0.160 (2)	0.779 (2)	0.058 (7)*	
H2N2	0.9324 (19)	−0.0897 (18)	0.8401 (18)	0.051 (6)*	
H1N3	0.9858 (19)	0.0747 (18)	0.7650 (18)	0.051 (6)*	
H1N4	0.414 (2)	0.6363 (17)	0.3130 (17)	0.053 (6)*	
H2N4	0.531 (2)	0.700 (2)	0.336 (2)	0.080 (8)*	
H3N4	0.4843 (19)	0.6587 (18)	0.224 (2)	0.055 (6)*	
H1N5	0.578 (2)	0.650 (2)	0.5188 (19)	0.057 (7)*	
H2N5	0.6724 (18)	0.5757 (16)	0.5834 (17)	0.042 (5)*	
H1N6	0.7491 (19)	0.4214 (18)	0.5080 (19)	0.055 (6)*	

### Atomic displacement parameters (Å^2^)

**Table:**

	*U*^11^	*U*^22^	*U*^33^	*U*^12^	*U*^13^	*U*^23^
Cl1	0.0468 (2)	0.0417 (3)	0.02393 (17)	−0.00571 (18)	0.00127 (15)	−0.00426 (15)
Cl2	0.0490 (2)	0.0429 (3)	0.02580 (18)	−0.00792 (19)	−0.00072 (16)	−0.00412 (16)
Cl3	0.0343 (2)	0.0577 (3)	0.02968 (19)	−0.00126 (19)	−0.00008 (15)	0.00466 (17)
Cl4	0.0431 (2)	0.0488 (3)	0.02839 (19)	0.0020 (2)	−0.00015 (15)	−0.00488 (17)
N1	0.0352 (7)	0.0293 (8)	0.0280 (7)	−0.0041 (6)	0.0027 (6)	−0.0052 (5)
N2	0.0442 (8)	0.0546 (11)	0.0233 (7)	−0.0027 (8)	−0.0026 (6)	0.0061 (6)
N3	0.0359 (7)	0.0422 (9)	0.0292 (7)	−0.0067 (6)	−0.0008 (6)	−0.0108 (6)
C1	0.0418 (10)	0.0318 (10)	0.0498 (10)	−0.0081 (8)	0.0052 (8)	−0.0057 (8)
C2	0.0479 (10)	0.0317 (10)	0.0419 (9)	0.0011 (8)	0.0043 (8)	0.0066 (7)
C3	0.0362 (8)	0.0341 (9)	0.0243 (7)	0.0026 (7)	−0.0023 (6)	0.0007 (6)
C4	0.0270 (7)	0.0254 (8)	0.0235 (6)	0.0011 (6)	0.0026 (5)	−0.0035 (5)
C5	0.0255 (7)	0.0347 (9)	0.0230 (6)	0.0019 (6)	0.0027 (5)	−0.0032 (6)
N4	0.0298 (7)	0.0355 (8)	0.0310 (7)	0.0013 (6)	−0.0010 (6)	0.0030 (6)
N5	0.0486 (9)	0.0462 (10)	0.0264 (7)	−0.0019 (8)	0.0008 (6)	−0.0056 (6)
N6	0.0358 (7)	0.0369 (8)	0.0282 (6)	0.0015 (6)	−0.0027 (5)	0.0064 (6)
C6	0.0371 (9)	0.0325 (10)	0.0448 (9)	0.0042 (8)	0.0043 (7)	0.0038 (7)
C7	0.0433 (9)	0.0323 (10)	0.0347 (8)	−0.0041 (8)	0.0079 (7)	−0.0036 (7)
C8	0.0343 (8)	0.0340 (9)	0.0250 (7)	−0.0056 (7)	0.0007 (6)	0.0004 (6)
C9	0.0255 (7)	0.0286 (8)	0.0256 (6)	−0.0034 (6)	0.0012 (5)	0.0041 (6)
C10	0.0276 (7)	0.0302 (8)	0.0242 (6)	−0.0045 (6)	0.0021 (5)	0.0021 (6)

### Geometric parameters (Å, °)

**Table:**

N1—C4	1.451 (2)	N4—C9	1.450 (2)
N1—H1N1	0.84 (2)	N4—H1N4	0.91 (2)
N1—H2N1	0.87 (2)	N4—H2N4	0.93 (3)
N1—H3N1	0.89 (2)	N4—H3N4	0.83 (2)
N2—C5	1.328 (2)	N5—C10	1.334 (2)
N2—H1N2	0.89 (2)	N5—H1N5	0.90 (2)
N2—H2N2	0.78 (2)	N5—H2N5	0.84 (2)
N3—C5	1.348 (2)	N6—C10	1.347 (2)
N3—C1	1.357 (2)	N6—C6	1.351 (2)
N3—H1N3	0.82 (2)	N6—H1N6	0.89 (2)
C1—C2	1.348 (3)	C6—C7	1.353 (2)
C1—H1A	0.90 (2)	C6—H6A	1.00 (2)
C2—C3	1.393 (3)	C7—C8	1.399 (2)
C2—H2A	0.94 (2)	C7—H7A	0.99 (2)
C3—C4	1.365 (2)	C8—C9	1.367 (2)
C3—H3A	0.857 (17)	C8—H8A	0.926 (16)
C4—C5	1.4145 (19)	C9—C10	1.4188 (19)
			
C4—N1—H1N1	111.6 (14)	C9—N4—H1N4	114.2 (14)
C4—N1—H2N1	109.7 (16)	C9—N4—H2N4	108.0 (15)
H1N1—N1—H2N1	106.9 (19)	H1N4—N4—H2N4	110 (2)
C4—N1—H3N1	112.3 (16)	C9—N4—H3N4	107.9 (15)
H1N1—N1—H3N1	117 (2)	H1N4—N4—H3N4	108.9 (18)
H2N1—N1—H3N1	98.5 (19)	H2N4—N4—H3N4	108 (2)
C5—N2—H1N2	122.6 (15)	C10—N5—H1N5	122.7 (14)
C5—N2—H2N2	122.3 (16)	C10—N5—H2N5	117.7 (14)
H1N2—N2—H2N2	114 (2)	H1N5—N5—H2N5	119 (2)
C5—N3—C1	123.65 (14)	C10—N6—C6	123.92 (14)
C5—N3—H1N3	120.3 (16)	C10—N6—H1N6	125.0 (14)
C1—N3—H1N3	116.0 (16)	C6—N6—H1N6	110.9 (14)
C2—C1—N3	120.59 (17)	N6—C6—C7	120.63 (17)
C2—C1—H1A	130.1 (14)	N6—C6—H6A	110.7 (12)
N3—C1—H1A	109.2 (14)	C7—C6—H6A	128.7 (12)
C1—C2—C3	118.52 (17)	C6—C7—C8	118.28 (16)
C1—C2—H2A	121.7 (13)	C6—C7—H7A	119.6 (11)
C3—C2—H2A	119.7 (13)	C8—C7—H7A	122.1 (11)
C4—C3—C2	120.41 (15)	C9—C8—C7	120.58 (14)
C4—C3—H3A	118.8 (13)	C9—C8—H8A	120.0 (11)
C2—C3—H3A	120.8 (13)	C7—C8—H8A	119.4 (11)
C3—C4—C5	120.64 (14)	C8—C9—C10	120.20 (15)
C3—C4—N1	120.47 (13)	C8—C9—N4	120.20 (13)
C5—C4—N1	118.84 (13)	C10—C9—N4	119.61 (14)
N2—C5—N3	119.72 (15)	N5—C10—N6	118.62 (15)
N2—C5—C4	124.16 (16)	N5—C10—C9	125.02 (16)
N3—C5—C4	116.12 (14)	N6—C10—C9	116.36 (14)
			
C5—N3—C1—C2	−1.9 (3)	C10—N6—C6—C7	0.6 (3)
N3—C1—C2—C3	−0.2 (3)	N6—C6—C7—C8	−0.3 (3)
C1—C2—C3—C4	0.5 (3)	C6—C7—C8—C9	1.0 (2)
C2—C3—C4—C5	1.0 (2)	C7—C8—C9—C10	−1.9 (2)
C2—C3—C4—N1	−176.49 (16)	C7—C8—C9—N4	177.80 (15)
C1—N3—C5—N2	−176.12 (17)	C6—N6—C10—N5	177.71 (16)
C1—N3—C5—C4	3.3 (2)	C6—N6—C10—C9	−1.4 (2)
C3—C4—C5—N2	176.56 (16)	C8—C9—C10—N5	−177.02 (15)
N1—C4—C5—N2	−5.9 (2)	N4—C9—C10—N5	3.3 (2)
C3—C4—C5—N3	−2.8 (2)	C8—C9—C10—N6	2.0 (2)
N1—C4—C5—N3	174.72 (14)	N4—C9—C10—N6	−177.64 (14)

### Hydrogen-bond geometry (Å, °)

**Table:**

*D*—H···*A*	*D*—H	H···*A*	*D*···*A*	*D*—H···*A*
N1—H1N1···Cl2^i^	0.84 (2)	2.26 (2)	3.081 (2)	164.4 (18)
N1—H2N1···Cl1^ii^	0.87 (2)	2.27 (2)	3.114 (1)	167 (2)
N1—H3N1···Cl4^iii^	0.89 (2)	2.47 (2)	3.223 (2)	143.2 (18)
N2—H1N2···Cl1^iv^	0.90 (2)	2.32 (2)	3.219 (2)	177.6 (16)
N2—H2N2···Cl2^v^	0.79 (2)	2.59 (2)	3.242 (2)	142 (2)
N3—H1N3···Cl2	0.82 (2)	2.26 (2)	3.060 (2)	166 (2)
N4—H1N4···Cl3^vi^	0.91 (2)	2.17 (2)	3.049 (2)	163.2 (17)
N4—H2N4···Cl4	0.93 (2)	2.77 (2)	3.454 (2)	131.5 (17)
N4—H2N4···Cl1^vi^	0.93 (2)	2.51 (2)	3.148 (2)	126.4 (18)
N4—H3N4···Cl4^vii^	0.83 (2)	2.30 (2)	3.128 (2)	175 (2)
N5—H1N5···Cl4	0.91 (2)	2.29 (2)	3.193 (2)	172.0 (18)
N5—H2N5···Cl1^viii^	0.84 (2)	2.77 (2)	3.340 (2)	126.6 (17)
N6—H1N6···Cl3	0.89 (2)	2.22 (2)	3.057 (1)	156.6 (19)
C7—H7A···Cl4^ix^	0.99 (2)	2.745 (19)	3.459 (2)	129.4 (14)

Symmetry codes: (i) −*x*+2, *y*−1/2, −*z*+3/2; (ii) −*x*+1, −*y*, −*z*+1; (iii) *x*, *y*−1, *z*; (iv) −*x*+1, *y*−1/2, −*z*+3/2; (v) −*x*+2, −*y*, −*z*+2; (vi) −*x*+1, −*y*+1, −*z*+1; (vii) *x*, −*y*+3/2, *z*−1/2; (viii) −*x*+1, *y*+1/2, −*z*+3/2; (ix) −*x*+1, *y*−1/2, −*z*+1/2.
